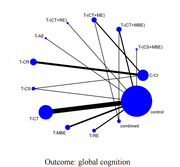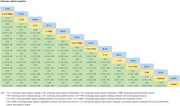# Effects of technology‐based cognitive and exercise interventions on cognitive function in persons with mild cognitive impairment: A systematic review, network meta‐analysis, and meta regression

**DOI:** 10.1002/alz.083781

**Published:** 2025-01-09

**Authors:** Yifan Ye, Lifeng Zhang

**Affiliations:** ^1^ School of Nursing, Sun Yat‐sen University, Guangzhou, Guangdong China

## Abstract

**Background:**

Technology has been increasingly integrated into controlling the decline of cognitive function for persons with mild cognitive impairment (MCI). It is unclear whether technology‐based cognitive and exercise interventions could generate synergistic benefits and what components would optimize this effect.

**Methods:**

In this study, we searched MEDLINE, Web of Science, Scopus, Embase and APA PsycInfo from inception to Nov 4, 2023. We included randomized controlled trails that evaluated the effects of technology‐based cognitive and exercise interventions on cognitive function for persons with MCI. Our primary outcome was global cognition. Outcomes were summarized in narrative synthesis and combined using meta‐analysis. Pairwise meta‐analysis and network meta‐analysis were sequentially performed to identify the effects of each category of interventions and their comparative intervention effectiveness, respectively. Meta‐regression was performed to examine any influence of study design and participants’ characteristics on the intervention effectiveness. This systematic review protocol was registered in PROSPERO (CRD 42023486359).

**Findings:**

A total of 28 studies with 1633 participants were included. The results of pairwise meta‐analyses indicated that technology‐based cognitive and exercise interventions were superior to active/passive controls in global cognition, cognitive shifting, proceeding speed, working memory, delayed recall, and category fluency (p<0.05). The results of network meta‐analyses indicated that the optimal components were cognitive stimulation combined with mind‐body exercise in improving global cognition (SUCRA: 77.0%, SMD 0.85, 95%CI ‐0.17 to 1.87) and cognitive shifting (SUCRA: 92.4%, SMD 1.57, 95%CI 0.88 to 2.25), while cognitive training combined with mind‐body exercise were the most beneficial in developing proceeding speed (SUCRA: 88.5%, SMD 0.68, 95%CI 0.14 to 1.22). Meta‐regression further suggested that the effects of the tested interventions were independent of the various factors relating to study design and participants’ characteristics.

**Interpretation:**

Technology‐based cognitive and exercise interventions can be effective in improving global cognition and core subdomains of cognition in persons with MCI. This review highlights the more superior effects of technology‐based cognitive stimulation combined with mind‐body exercise in improving global cognition.